# The clinical correlates of vaccine-induced immune thrombotic thrombocytopenia after immunisation with adenovirus vector-based SARS-CoV-2 vaccines

**DOI:** 10.1093/immadv/ltab019

**Published:** 2021-08-17

**Authors:** Eleanor R Gaunt, Neil A Mabbott

**Affiliations:** The Roslin Institute & Royal (Dick) School of Veterinary Studies, University of Edinburgh, Easter Bush, Midlothian EH25 9RG, UK

**Keywords:** SARS-CoV-2, COVID-19, coronavirus, vaccination, heparin-induced thrombocytopenia, vaccine-induced immune thrombotic thrombocytopenia

## Abstract

We are at a critical stage in the COVID-19 pandemic where vaccinations are being rolled out globally, in a race against time to get ahead of the SARS-CoV-2 coronavirus and the emergence of more highly transmissible variants. A range of vaccines have been created and received either emergency approval or full licensure. To attain the upper hand, maximum vaccine synthesis, deployment, and uptake as rapidly as possible is essential. However, vaccine uptake, particularly in younger adults is dropping, at least in part fuelled by reports of rare complications associated with specific vaccines. This review considers how vaccination with adenovirus vector-based vaccines against the SARS-CoV-2 coronavirus might cause rare cases of thrombosis and thrombocytopenia in some recipients. A thorough understanding of the underlying cellular and molecular mechanisms that mediate this syndrome may help to identify methods to prevent these very rare, but serious side effects. This will also help facilitate the identification of those at highest risk from these outcomes, so that we can work towards a stratified approach to vaccine deployment to mitigate these risks.

## Introduction

Currently in the UK (August, 2021), relatively low numbers of patients with severe disease, hospitalisations and deaths after infection with severe acute respiratory syndrome corona virus 2 (SARS-CoV-2) have been attained in recent weeks, largely attributable to a phenomenal vaccine deployment effort. However, an emerging problem is vaccine hesitancy [1]. A contributing factor towards this is the association of vaccinations, specifically those using an adenovirus-based delivery system, with rare instances of severe outcomes including clotting syndromes that have been collectively referred to as vaccine induced immune thrombotic thrombocytopenia (VITT). In this review, we consider how adenovirus vector-based vaccines, including those against SARS-CoV-2, might cause rare cases of thrombosis and thrombocytopenia in some recipients. We introduce the relevant biology underpinning development of these vaccines, describe analogous clotting syndromes including heparin-induced thrombocytopenia (HIT) and compare VITT with HIT. Finally, we emphasise the importance of the continued use of adenovirus-based vaccines. Detailed analysis of the underlying cellular and molecular mechanisms that mediate this syndrome may help to identify methods to identify those at higher risk of these outcomes and ultimately prevent these very rare, but serious side effects.

Several different vaccines have been produced in response to the SARS-CoV-2 pandemic. The most common immunogenic approach used in the vaccines developed so far has been based on the induction of specific immunity to the SARS-CoV-2 spike glycoprotein antigen, using a range of platforms for its delivery. Several spike-targeting vaccines have successfully passed safety and efficacy trials, and these have received emergency approval and licensing for use in many countries (Table 1). The development, approval and implementation of these vaccines have been undertaken at an astonishing pace. Indeed, by 27 April 2021, just 16 months since the first infections with the SARS-CoV-2 coronavirus were identified [2], over a billion vaccine doses had been administered to 570 million individuals around the world [3].

**Table 1.** SARS-CoV-2 vaccines with full licensure in multiple countries

Vaccine name(s)	Manufacturer	No. of countries with full (emergency) licensure ^a^	Date of first use in UK	Delivery system	Delivery details	Serious side effects (all very rare)	Refs
ChAdOx1 nCov-19/ Vaxzevria/ Covishield/ AZD1222	Astra Zeneca	2 (170)	Jan 2021	Vehicle	Chimpanzee AdV	Anaphylaxis, thrombosis with thrombocytopenia	[4]
BNT162b2	Pfizer-BioNTech	5 (108)	Dec 2020	mRNA	mRNA and lipid nanoparticles	Anaphylaxis. myocarditis or pericarditis	[5, 6]
mRNA-1273	Moderna	2 (74)	Apr 2021	mRNA	mRNA and lipid nanoparticles	Anaphylaxis, seizures, myocarditis or pericarditis	[5, 7]
BBIBP-CorV	Sinopharm	4 (73)	Not expected	Inactivated	Chemically inactivated virus	None reported	[8]
Ad26.COV2.S	Johnson and Johnson / Jannsen	2 (77)	Pending; emergency approval May 2021	Vehicle	AdV 26 (human)	Hypersensitivity not classified as anaphylaxis, blood clots	[9, 10]
Gam-COVID-Vac/ Sputnik V	Gamaleya	2 (72)	Not expected	Vehicle	AdV 26 then AdV 5 (both human)	Deep vein thrombosis, haemorrhagic stroke, hypertension / insufficiently reported	[11, 12]

^a^Updated data on COVID-19 vaccinations available here [13].

At the time of writing (August 2021), the most frequently used SARS-CoV-2 vaccines are based on mRNA or adenovirus vector systems that deliver genetic material encoding the coronavirus spike protein into cells. These include the BNT162b2 (Pfizer–BioNTech) [14] and mRNA-1273 (Moderna) [15] vaccines that contain mRNA encoding the coronavirus spike glycoprotein encased in lipid nanoparticles. These mRNA-based vaccines represent the first licensed vaccines using this type of platform.

The ChAdOx1 nCov-19 (Oxford-AstraZeneca) vaccine, in contrast, comprises a non-pathogenic recombinant, replication-deficient chimpanzee Ad5 adenovirus vector (originally called ChAdY25 and later renamed ChAdOx1; [16]) encoding the spike glycoprotein [17]. The Ad26.COV2.S (Johnson & Johnson/Janssen) vaccine similarly utilises a human replication-deficient Ad26 adenovirus-based vector for spike protein delivery [18]. Adenovirus vectors are also used in the Gam-COVID-Vac (Sputnik V, Gamaleya) vaccine, but in this instance they are applied in a heterologous prime/boost approach. An Ad26 vector encoding the spike glycoprotein is used for the first (prime) injection, and this is followed by a booster injection comprising a spike glycoprotein-encoding human-derived Ad5 vector [11].

It was possible to conceptualise and produce the ChAdOx1 nCoV-19 vaccine so quickly because the same platform had previously been used for the development of a vaccine targeting Middle Eastern Respiratory Syndrome (MERS)–CoV. The ChAdOx1 MERS vaccine provided protective immunity to MERS-CoV infection in rhesus macaques [19], and was deemed safe in Phase I clinical trials in humans [20]. The rapid application of this platform to target SARS-CoV-2 instead was therefore straightforward; this vaccine was designed over a weekend [21]. In the ChAdOx1 nCoV-19 vaccine, the Ad5 vector has been engineered to also encode the full-length SARS-CoV-2 spike glycoprotein downstream of a tissue plasminogen activator leader sequence that directs the target protein into the secretory pathway, under a CMV promoter. The spike sequence was also codon optimised for more efficient translation [17, 22]. The CHAdOx1 vector has been rendered replication incompetent through deletion of the E1 and E3 genes [23].

For the ChAdOx1-nCoV vaccine, a dose of 5 × 10^10^ viral particles (corresponding to the highest dose of ChAdOx1 MERS tested in humans) is administered by intramuscular injection during both the first and second vaccinations. An error in virus quantification during clinical trials resulted in second doses being tested at both full and half dose regimen [24]. Lower antibody titres were seen in individuals boosted with the half-dose compared with full dose, and no differences in side effects were detected between individuals given a first or second injection at half dose, compared with those given standard doses at both times [24, 25].

The rapid deployment and widespread uptake of the described vaccines has had a dramatic impact in reducing the incidence of SARS-CoV-2 infections, transmissions, and associated serious illnesses, especially in countries such as Israel and the UK where large proportions of adults have been vaccinated [26–28]. A study of more than 365,000 UK households released in April 2021 reported that the ChAdOx1 nCov-19 and BNT162b2 vaccines were effective in reducing SARS-CoV-2 transmissions to others in the same household by 40–50% even after a single dose [26], although vaccine efficacy is lower against the rapidly emerging delta SARS-CoV-2 variant [29, 30]. Analysis by Public Health England published in July 2021 has also estimated that the UK COVID-19 vaccination programme had prevented between 21.3 and 22.9 million infections and 57,500–62,700 deaths [31].

A large UK community cohort study revealed that side effects after vaccination with either a single dose of the BNT162b2 or ChAdOx1 nCoV-19 vaccines were experienced in <30% of recipients, and these were typically mild and short-lived [32]. In these instances the most-commonly reported side effects included a sore arm at the injection site, headache and systemic flu-like symptoms lasting approximately 1–2 days. Similar findings were reported in clinical trials using the Gam-COVID-Vac vaccine [11]. This was consistent with the side effects observed during clinical trials. However, following the administration of these vaccines to millions of recipients around the world some extremely rare but serious instances of potentially vaccine-induced side effects have been reported. These include reports of thrombosis (blood clots) accompanied by depletion of platelets (thrombocytopenia) within 5–24 days after injection with either the ChAdOx1 nCov-19 or the Ad26.COV2.S adenovirus-vector-based vaccines [9,33–35]. For the ChAdOx1 nCoV-19 vaccine, the Medicines and Healthcare products Regulatory Agency (MHRA) in the UK estimated the incidence of thromboembolic events accompanied by thrombocytopenia to be 14.9 per million first (or unknown) doses [36, 37]. For Ad26.COV2.S, six cases of cerebral venous sinus thrombosis (CVST) with thrombocytopenia, one of which was fatal, were reported in 7.2 million vaccinees [38]. This novel syndrome of combined thrombosis and thrombocytopenia observed in these vaccine recipients has been termed VITT [39]. Very rare instances of thrombocytopenia and bleeding but without thrombosis were also reported after injection with the BNT162b2 and mRNA-1273 mRNA-based vaccines, with 17 cases reported in over 20 million vaccinees [40]. The BNT162b2 and mRNA-1273 vaccines have also been associated with other very rare side effects including allergic reactions and anaphylaxis [41, 42], and myocarditis or pericarditis [5] (Table 1).

Among the patients that had received adenovirus-based SARS-CoV-2 vaccines and experienced clotting shortly thereafter, the majority had thromembolic events in unusual locations in the body. These events included cerebral venous sinus thrombosis (CVST in the brain), splanchnic vein thrombosis (abdomen) and hepatic vein thrombosis (liver). These patients also presented with moderate to severe thrombocytopenia, and some unfortunately died. The incidence of CVST in adults in the general population is extremely rare (approximately 1% of all stroke forms), with estimates suggesting there are between 2 and 15.7 cases annually per million individuals [43–46]. The similarly rare incidence of these cases of thrombosis in combination with thrombocytopenia in UK recipients of the ChAdOx1 nCov-19 vaccine [36] makes identifying a definitive link with vaccination difficult. However, the available evidence suggests this should be considered a possible, but very rare side effect of these vaccines. This assessment has prompted some medical health agencies around the world to take a cautious approach and update their safety guidance, and in some instances, to halt the use of adenovirus vector-based SARS-CoV-2 vaccines, or restrict their use to certain age groups [36].

## Vaccines and viruses associated with clotting

Many viral infections including SARS-CoV-2 can themselves induce thrombocytopenia [47, 48]. Additionally, potential links between the use of vaccines (other than those targeting SARS-CoV-2) and rare instances of thrombocytopenia have been described. The best-evidenced association is that of the live measles, mumps and rubella vaccines with thrombocytopenic outcome reported to occur in 1 in 21,000 to 1 in 40,000 vaccine recipients [49, 50]. However, it is important to stress that this rate is considerably lower than the occurrence of thrombocytopenia following natural infection with measles or rubella [51]. Thrombocytopenia has also long been recognised as a potentially serious adverse reaction following use of adenovirus-based DNA vectors [52]. Nevertheless, for the vast majority of individuals the benefits of using adenovirus vector-based vaccines to provide protection against the hospitalisation and death that COVID-19 can cause, far out-weigh the risks associated with these very rare side effects. Clotting events are also far more likely in COVID-19 patients than as a result of inoculation with the vaccines developed against this disease. For example, clotting outcomes in COVID-19 patients admitted to intensive care units in the Netherlands occurred in approximately 49% of patients [53, 54].

These rare cases of vaccine-associated thrombosis accompanied with thrombocytopenia have (so far) only been reported in recipients within 5 to 24 days of injection with the ChAdOx1 nCov-19 or Ad26.COV2.S adenovirus vector-based vaccines, and not after injection with mRNA-based vaccines [33, 55]. This implies that there may be features of these vaccines, independent of the coronavirus spike glycoprotein antigen that can trigger these serious side effects in extremely rare circumstances.

## Mechanistic insights into adenovirus-vector-associated clotting events

In order to understand the possible mechanisms underpinning VITT, let us first briefly consider the sequence of events following vascular injury that lead to the formation of a clot (thrombosis). Clotting in response to vascular injury and bleeding is an essential process of tissue repair known as haemostasis. Vascular injury causes vasoconstriction (to reduce blood flow) and exposes collagen at the lesion site. This induces the adherence, activation and aggregation of platelets that form a platelet plug in order to seal off the lesion in the injured vessel [56]. von Willebrand factor (vWF) constitutively secreted by platelets and endothelial cells adheres to the exposed collagen and mediates the recruitment and adhesion of platelets to the lesion site. Multiple signalling cascades then converge to result in cross-linking of vWF and/or fibrinogen to receptors on platelets, resulting in their activation. Platelet activation leads to changes in their shape, which stimulates the release of their secretory granules. This triggers the production of further coagulation factors and the activation of multiple signalling cascades that converge on the formation of thrombin. The generated thrombin then mediates the conversion of circulating fibrinogen into an insoluble fibrin network that helps to strengthen the platelet plug. Although an essential process to help repair injured blood vessels, clots can cause pathology by restricting blood flow through the affected tissue.

Platelets are highly granular, and upon activation (e.g. during a clotting response) release the contents of a specific subset of granules termed ‘α-granules’ into the circulatory system. Of the hundreds of different α-granule constituents, one of the most abundant factors released during platelet activation is chemokine (C-X-C motif) ligand 4 (CXCL4), also known as platelet factor 4 (PF4). Aside from its roles in clotting pathways, PF4 is involved in inter-cellular innate immune signalling, including various activities such as activating neutrophil degranulation, monocyte recruitment and facilitating monocyte differentiation into macrophages. PF4 has been suggested as a pivotal factor that mediates the aberrant clotting events that have occurred after immunisation with adenovirus-vector-based vaccines [34, 57].

Platelet factor 4 is positively charged (cationic) and can complex with negatively charged (polyanionic) molecules such as heparin and glycosaminoglycans (GAGs) present in blood, forming complexes on the surfaces of platelets [58]. The first hints at a possible mechanistic link between adenovirus-based vaccination and clotting outcomes came from the analysis of sera from a cohort of German and Austrian patients that developed moderate-to-severe thrombocytopenia with unusual thromboses 5–16 days after immunisation with the ChAdOx1 nCov-19 vaccine. Each candidate in the study had sera with high-titres of antibodies (Abs) that could bind to cationic PF4 in complex with other polyanionic molecules [34, 39]. Subsequently, a similar association was also reported in a small number of recipients of the Ad26.COV2.S vaccine [9, 38, 59].

## Parallels with heparin-induced thrombocytopenia

### The central role of PF4 in platelet activation and clearance

In its regular morphology PF4 is not immunogenic. However, when PF4 is released from platelets (Fig. 1.1) it can bind to heparin or other polyanionic molecules and form ‘ultra-large’ complexes (Fig. 1.2). This aggregation causes conformational changes to PF4 which exposes epitopes that can induce the formation PF4-heparin/polyanion-specific Abs (Fig. 1.3), and these can activate platelets via the binding of Abs to Fc fragment of IgG receptor IIa (FcγRIIa) expressed on their surfaces (Fig. 1.4). The PF4-heparin ultra-large complexes also activate the complement system leading to their opsonisation by complement components C3 and C4 (Fig. 1.3). This mediates the uptake and retention of these complement-opsonised complexes by complement receptor 2 (CR2/CD21)-expressing B cells [61]. The PF4-heparin-containing complexes are then delivered to B cell follicles within secondary lymphoid organs where anti-PF4-heparin-specific Abs are produced.

**Figure 1.**
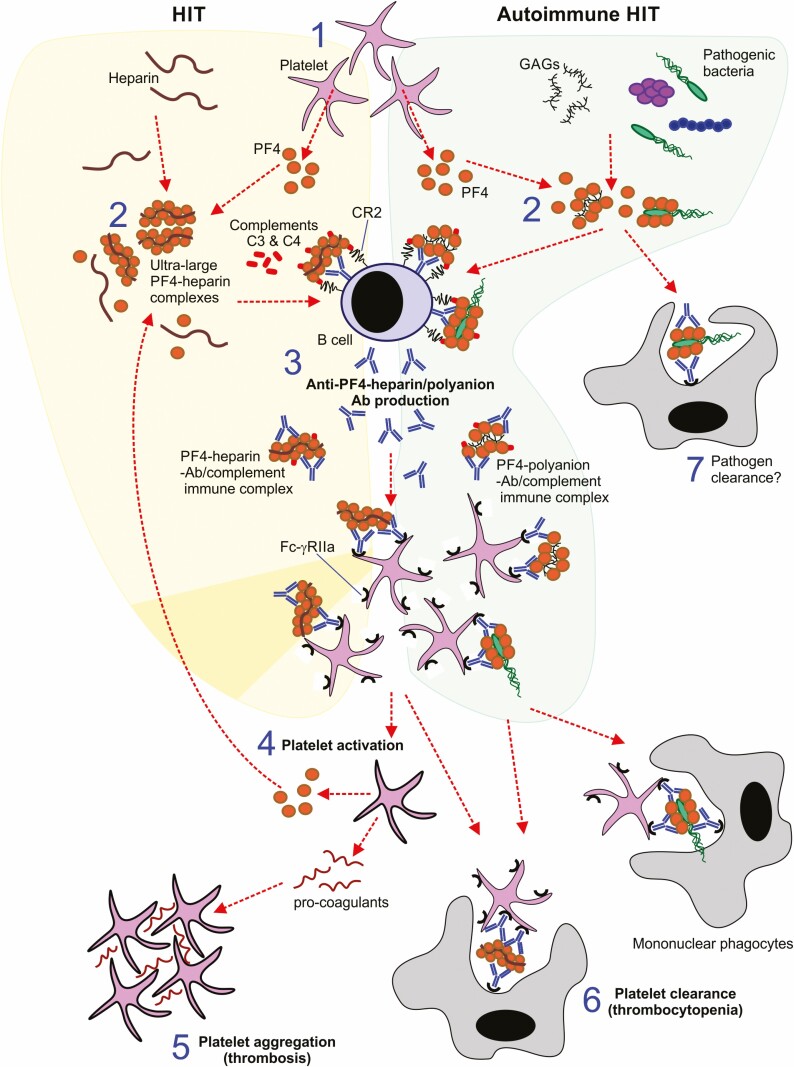
The central role of PF4 in heparin-induced thrombocytopenia (HIT) and autoimmune HIT. 1, Activated platelets release PF4. 2, PF4 is cationic and can bind to heparin or other polyanionic molecules (autoimmune HIT) and form ‘ultra-large’ complexes. 3, The binding of PF4 to heparin/polyanions exposes epitopes in PF4 that induce the formation PF4-heparin/polyanion-specific Abs. 4, Binding of PF4-heparin/polyanion-Ab immune complexes to Fcγ receptor IIa (FcγRIIa) on the platelet surface leads to their activation, creating a positive feedback-loop whereby PF4 released from activated platelets amplifies the production of anti-PF4-heparin-specific Abs. 5, A severe prothrombotic state is ultimately created. 6, Thrombocytopenia also occurs due to increased removal of platelets by mononuclear phagocytes. 7, Since PF4 can bind to certain pathogenic microorganisms it is plausible that this may play a role in protection against infection by aiding their clearance by mononuclear phagocytes.

Platelet activation triggers the release of more PF4, further enhancing the formation of PF4-heparin/polyanion-containing complexes (Fig. 1.4 → 1.2) [62]. This creates a positive feedback-loop whereby PF4 released from activated platelets amplifies the production of anti-PF4-heparin-specific Abs. During this process, Abs with particularly strong binding may be generated that can bind PF4 in the *absence* of polyanions – i.e. PF4-specific autoantibodies [63].

The combined actions of platelets and mononuclear phagocytes leads to the development of a severe prothrombotic state (Fig. 1.5) that is accompanied by increased removal of platelets (thrombocytopenia) (Fig. 1.6); this can be lethal [64].

### The central role of PF4 in endothelial cell damage

During HIT, the increased availability of PF4 can also activate endothelial cells (Fig. 2.1), and this can stimulate the recruitment and activation of monocytes and neutrophils. For example, anti-PF4 Abs from HIT patients can bind to PF4-complexed with GAGs on the surfaces of activated endothelial cells (Fig. 2.2), especially microvascular endothelial cells [65, 66]. This can cause further activation and damage to endothelial cells accompanied by the release of tissue factor, thrombodulin, and vWF – the latter of which can also bind PF4 (Fig. 2.3) [65–67]. A positive feedback loop is similarly created whereby the enhanced formation of Ab-PF4-GAG-containing immune complexes cause further activation and injury to local endothelial cells (Fig. 2.4), indicating that microvasculature damage may contribute to the development of HIT.

**Figure 2.**
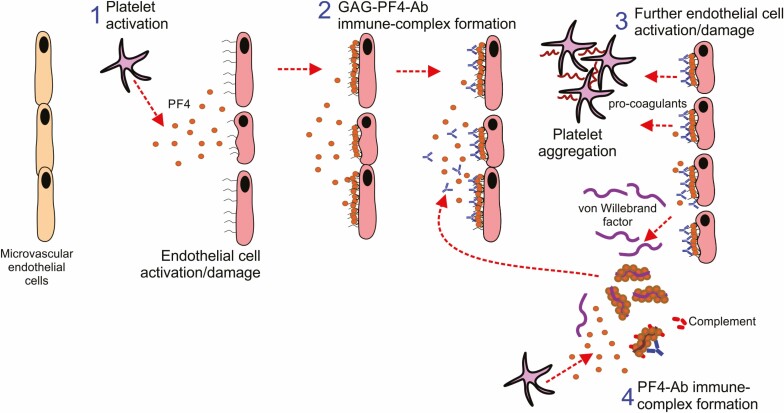
The central role of PF4 in endothelial cell damage during HIT. 1, PF4 can activate local endothelial cells. 2, PF4 can complex with GAGs on the surfaces of activated endothelial cells. 3, This can cause further activation and damage to the endothelium and the release of vWF and pro-coagulants. 4, A positive feedback loop can be created whereby the enhanced formation of Ab-PF4-GAG-containing immune complexes leads to further activation and damage the endothelium.

### The central role of PF4 in monocyte activation and thrombin-mediated platelet aggregation

The release of PF4 from activated platelets during HIT can also stimulate the recruitment of monocytes (Fig. 3.1). Here, PF4 also complexes with the GAG side-chains on the surfaces of monocytes (Fig. 3.2). The binding of anti-PF4 Abs to these PF4-GAG-containing complexes can then activate the monocytes via FcγRIIa stimulating the production of pro-coagulant factors, tissue factor and interleukin-8 (IL-8) (Fig. 3.3) [68–70]. Activation-induced changes to the GAGs on the monocyte surface further enhance this response [69]. The pro-coagulant activity of the monocytes generates thrombin (Fig. 3.4), which can contribute to HIT through the trans-activation and consequent aggregation of platelets [70].

**Figure 3.**
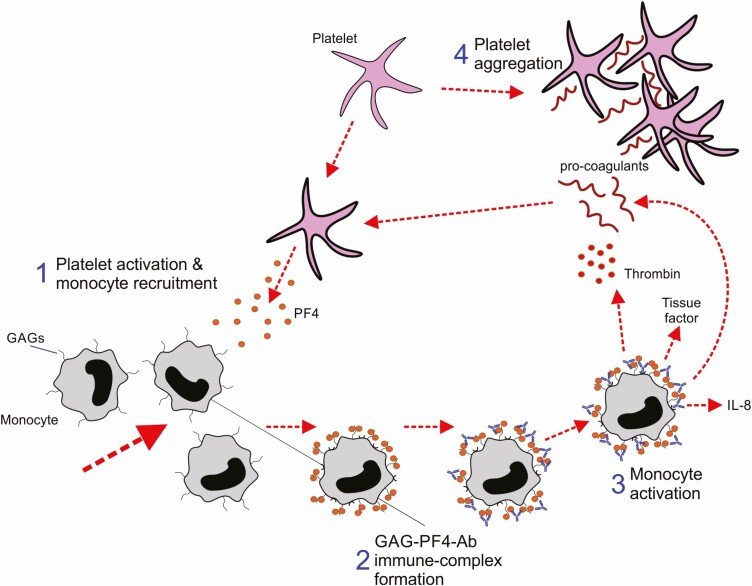
The central role of PF4 in monocyte activation during HIT. 1, PF4 released from platelets during HIT can activate and recruit monocytes. 2, Complexes can form between PF4 and the GAG side-chains on monocyte surfaces. 3, PF4-GAG-Ab containing immune complexes can activate monocytes via FcγRIIa. 4, The pro-coagulant activity of the monocytes can trigger a positive feedback loop leading to further platelet activation and aggregation.

### The central role of PF4 in NETosis

Neutrophils provide an important first line of defence in the clearance of invading pathogens. Neutrophil activation is typically induced via the binding of Abs to Fcγ receptors on their cell surfaces, but they can also be activated via stimulation from platelet-derived P-selectin. Physical interactions between neutrophils with platelets (Fig. 4.1), or Ab-mediated stimulation via Fcγ receptors (including PF4-heparin-Ab complexes; Fig. 4.2) trigger the release of fibrous complexes from neutrophils called neutrophil extracellular traps (NETs) through a process known as NETosis (Fig. 4.3). These complexes comprise DNA and associated proteins that ‘trap’ extracellular pathogens and destine them for phagocytosis [71]. While the release of NETs from neutrophils may provide protection against infection with certain pathogenic microorganisms, their excessive formation can contribute to the pathology of some inflammatory and autoimmune diseases [72]. NETs could also play an important role in the development of the thrombosis in HIT [73], since neutrophils can be activated after *in vitro* treatment with Abs specific for PF4 [71]. Immune complexes containing heparin, PF4, and Abs can activate NETosis in neutrophils either directly via stimulation of their FcγRIIa by anti-PF4 Ab, or indirectly via the FcγRIIa-mediated activation of platelets [73]. The large amounts of negatively charged DNA within these NETs may then complex with PF4 (Fig. 4.4), enhancing platelet activation and coagulation and the production of anti-PF4-heparin Abs [74] (Fig. 4.4 → Fig. 4.1).

**Figure 4.**
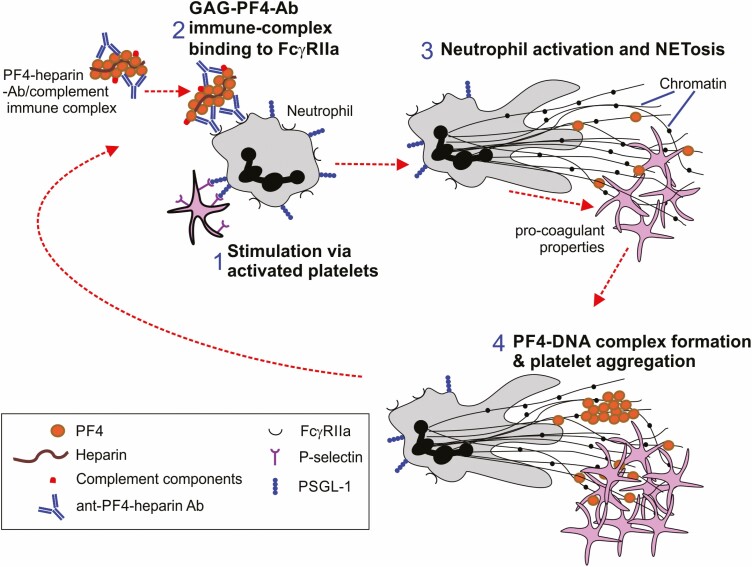
The central role of PF4 in NETosis during HIT. 1, Interactions between neutrophils and platelets, or 2, Ab-mediated stimulation via Fcγ receptors (including PF4-heparin-Ab complexes) can each activate neutrophils. 3. This can stimulate the release neutrophil extracellular traps (NETs), known as NETosis. NETs contain DNA and proteins that ‘trap’ extracellular pathogens and destine them for phagocytosis. 4, The negatively charged DNA within these NETs may then trap and complex with PF4, enhancing platelet activation and coagulation and the production of anti-PF4-heparin Abs, resulting in a positive feedback loop.

### The central role of PF4 in pathogen clearance

Many individuals, including children <6 months old [75], have PF4-heparin-specific B cells, and so it is reasonable to expect that they have a beneficial role that outweighs their involvement in the development of HIT. For example, the binding of PF4 to the polyanionic surfaces of the Gram positive bacteria *Staphylococcus aureus*, *Streptococcus pneumoniae* and *Listeria monocytogenes* or the Gram-negative bacteria *Escherichia coli* and *Neisseria meningitidis* may provide innate immune protection by mediating their removal from the circulation by natural PF4-heparin-specific Abs [75, 76] (Fig. 1.7). Nevertheless, although the presence of PF4-heparin-specific B cells may be relatively common, the prevalence of HIT is not, indicating that in rare cases the dysregulation of this activity may lead to HIT. It is unclear what events lead to this dysregulation, but the availability of PF4 antigen is central to the process [77].

## How might vaccination cause VITT?

HIT and VITT share several common pathological features. The sera from individuals diagnosed as potential VITT patients that had received the ChAdOx1 nCov-19 vaccine could activate platelets in the presence of PF4 (as in Fig. 1.4), and this was inhibited by the addition of a blocking Fcγ-RIIa-specific Ab [39]. These characteristics share notable similarities with HIT, whereby the binding of anti-PF4 Abs to complexes of PF4 and polyanionic heparin, activates platelets via their FcγRIIa, ultimately leading to thrombosis and thrombocytopenia (Fig. 1) [64]. However, in contrast to patients with HIT, none of the patients that developed thrombosis and thrombocytopenia post-vaccination were known to have been treated with heparin, and the anti-PF4 IgG Abs in their sera could bind PF4 in the absence of heparin.

The induction of HIT by polyanionic factors other than heparin is known as autoimmune HIT, and cases of autoimmune HIT accompanied by CVST have been described [78]. Clinically diverse examples of autoimmune HIT-like syndromes occurring without prior exposure to exogenous heparin have been reported following: (i) infection with pathogens such as influenza A virus, HIV-1, SARS-CoV and SARS-CoV-2 [78–84]; (ii) treatment with polyanionic drugs such as pentosan polysulphate [85]; or (iii) in response to exposure to joint cartilage-associated GAGs released as a consequence of tissue damage during orthopaedic surgery [86]. As with heparin, polyanionic molecules produced in the above examples can similarly bind to PF4 and cause conformational changes to PF4 that expose epitopes that can induce the formation PF4-polyanion-specific Abs.

The ChAdOx-1 vaccine recipient VITT patient-derived anti-PF4 Abs did not recognise the coronavirus SARS-CoV-2 spike protein in *in vitro* assays, suggesting that it was unlikely that VITT was simply a consequence of cross-reactivity of the humoral immune response to the spike glycoprotein. A pre-print study from Greinacher and colleagues [57] has attempted to gain insight into the underlying mechanisms that may cause VITT. They used methods including 3D-super-resolution microscopy, transmission electron microscopy, mass spectrometry and nuclear magnetic resonance spectroscopy to investigate the interactions between components of the ChAdOx1 nCoV-19 vaccine and the sera of VITT patients. From preliminary data obtained they proposed a sequence of post-vaccine inflammatory responses in the VITT patients reminiscent of autoimmune HIT that may have triggered the induction of anti-PF4 Abs that caused prothrombotic reactions and thrombocytopenia. Independent studies in mice have also shown that intravenous injection with a recombinant replication-deficient Ad5 adenovirus vector (similar to that used in the ChAdOx1 nCov-19 vaccine and used in the second dose of the Gam-COVID-Vac) can trigger platelet activation and aggregation [52, 87]. The Ad5 vector also stimulated the release of ultra-large vWF from endothelial cells. These events could potentially cause thrombosis due to enhanced platelet aggregation, and thrombocytopenia due to the removal of platelets from the circulation by mononuclear phagocytes [52].

Greinacher and colleagues suggested that VITT is first initiated through interactions between the Ad5 vaccine vector and platelets, triggering their activation [57]. The activated platelets then release PF4 which can complex with polyanionic vaccine components, in a similar manner to the PF4-polyanionic complexes in autoimmune HIT patients (Fig. 1, green panel). The presence of these vaccine component-PF4 complexes in the circulation might then stimulate an acute pro-inflammatory response and the generation of anti-PF4 Abs, via a process analogous to HIT [64]. Exacerbated prothrombotic reactions were then proposed to occur in the blood-stream of VITT patients when PF4 is recognised and clustered on the surface of platelets by high avidity anti-PF4 Abs [57]. This, coupled with the opsonisation of the Ab-vaccine component-PF4 complexes by complement components, could then mediate the removal of platelets by mononuclear phagocytes, resulting in thrombocytopenia.

The polyanionic vaccine component/s that might bind platelets in certain individuals, and/ or complex with PF4 to induce the generation of anti-PF4 Abs are not known. It is possible that vaccine constituent polyanionic protein(s) play a role. Proteomics analyses suggested that the vaccine preparation contained a mixture of >1000 proteins including cell culture-derived human proteins and adenovirus vector proteins in addition to the SARS-CoV-2 spike glycoprotein [57]. The negatively charged DNA within the adenovirus vector might be also a credible target, since free nucleic acid (DNA and RNA) can bind to PF4 and trigger a conformational change, exposing immunogenic epitopes that may be recognised by anti-PF4 Abs. Complexes containing DNA and PF4 can also trigger the generation of anti-PF4-heparin Abs in mice [74]. However, the DNA in these vaccines is packaged within adenovirus vector particles, so it is uncertain whether sufficient free DNA would be available (for example due to virus particle lysis) in the serum to complex with PF4. Extracellular RNA is a possible alternative; while RNA is generally considered less stable than DNA, circular RNAs released in extracellular vesicles are highly stable and abundant in cell culture [88]. Greinacher and colleagues also suggested that ingredients in the vaccine carrier solution such as EDTA might cause capillary leakage [57], enhancing endothelial cell activation and damage, and leading to amplification of the acute inflammatory response to vaccination at the injection site.

Excessive NETosis has been associated with the development of thrombosis after virus infections, including SARS-CoV-2 [89], but it is unknown whether NETosis contributes to VITT. As described above (Fig. 4), NETs can stimulate the production of anti-PF4 Abs and have pro-coagulant properties that can contribute to the development of thrombosis in HIT [73, 74]. NETosis can also be triggered in neutrophils after *in vitro* treatment with PF4 and serum from VITT patients [57]. Extracellular DNases can degrade the DNA within these NETs, and this may help regulate the balance between NETosis and NET clearance to prevent excessive inflammation and pathology [90]. It was noteworthy that the sera of some VITT patients contained much lower levels of DNase activity when compared to healthy controls, and so it is possible that NET clearance was diminished in VITT patients [57]. This raises the hypothesis that dysregulated NETosis may contribute to the development of VITT.

## Why only some individuals?

**Figure 5.**
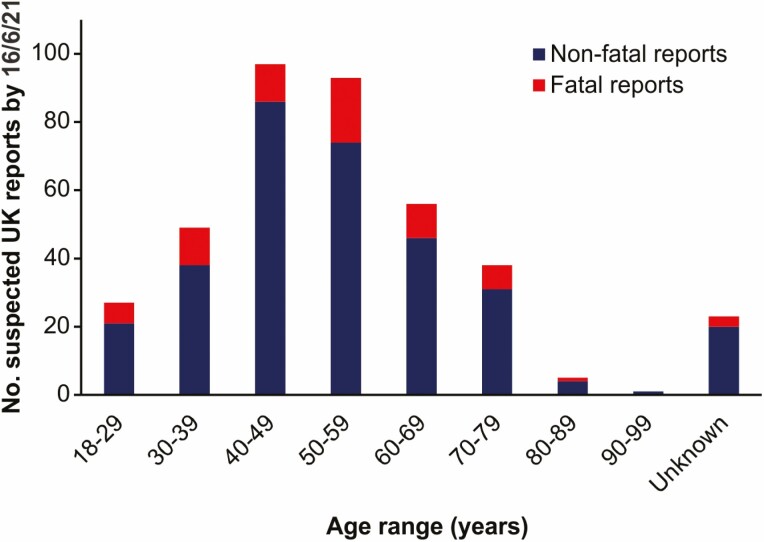
Numbers of suspected reports of thrombotic thrombocytopenia in the UK after immunization with the ChAdOx1 nCov-19 vaccine. Data presented by patient age group and include reported cases up to, and including, 16/6/21. Data derived from Ref. (91). Blue bars, non-fatal reports; Red bars, fatal reports.

In order to understand the events leading up to VITT, key information may be forthcoming from the identification of patient risk factors. Fortunately, the incidence of VITT in recipients of adenovirus-vector-based COVID-19 vaccinations has been extremely rare [36], and at the time of writing it was uncertain what characteristic/s in the VITT patients were responsible for their increased risk of developing these rare complications; there is no obvious group that has been disproportionately affected. The most comprehensive demographic data has been accrued by the UK ‘yellow card’ reporting system which catalogues the reported adverse reactions to the COVID-19 vaccines, although these are not independently verified [91]. As of 16 Jun 2021, 24.5 million first doses and 19.6 million second doses of the ChAdOx-1 nCoV-19 vaccine had been administered in the UK. Following vaccination, a total of 389 cases of thrombotic thrombocytopenia had been reported (8.8 cases per million doses). Of these, 203 cases were in females and 183 were in males, and 3 cases did not have a gender assigned. The proportion of vaccinations given to each gender group was not described. Amongst these 389 cases, 31 occurred after a second dose. The number of reported cases of thromboembolic events accompanied by thrombocytopenia in a range of age groups is shown in Fig. 5, however the total number of recipients in each age category was not provided. Of the affected patients, 68 had unfortunately died (17%), of whom 39 were female (19.2% of affected females) and 29 were male (15.8% of affected males). Additionally, CVST was reported in 140 vaccine recipients and other thromboembolic events were reported in 249 vaccine recipients.

If we make the assumption that the vaccinations were administered to approximately equal numbers of men and women, it would appear that the incidence of VITT is slightly higher in women than in men. This correlates with other non-vaccine-associated types of thromboses. There is an increased incidence of CVST in women of child-bearing age, during pregnancy and the post-partum period, and in those using oral contraceptives or oestrogen replacement therapies [92, 93]. Additional risk factors for CVST and HIT include the presence of high titre and affinity anti-PF4 antibodies, age (below 50 years old), obesity, tissue trauma, certain infections and genetics [64, 78, 84, 86, 92–95]. However, COVID-19 disease is itself associated with a higher risk of developing CVST [96] and HIT [97], and thrombocytopenia and thrombosis are relatively common in critically ill SARS and COVID-19 patients [81–83, 96–99].

Polymorphisms in specific genes involved in the HIT response can affect susceptibility to this outcome. For example, the risk of developing HIT is increased in patients that are homozygous for the FcγRIIIa-158VV allele, and these patients tend to have high levels of anti-PF4 Abs in their sera [100]. A similar association has been reported for the FcγRIIa-131RR allele [101–104]. In HIT patients these polymorphisms in FcγR-encoding genes might increase cell activation upon binding to Ab-PF4-heparin immune complexes [102]. PECAM-1 is also expressed on the surfaces of platelets and neutrophils, and the PECAM-1 125VV polymorphism is similarly associated with an increased risk of HIT [105]. A higher prevalence of the human platelet antigen (HPA)-1 a/b genotype among HIT patients has also been described, with the risk of developing thromboembolic complications increasing 8-fold in those carrying each of the FcγRIIA-131RR, and PECAM-1 VV125 polymorphisms and HPA-1 a/b genotype [105]. Polymorphisms in other genes such as *CXADR* (encoding the coxsackie-adenovirus receptor) could also contribute, for example by enhancing the affinity of the adenovirus vector for platelets [52, 106]. None of these genotypes have as yet been associated with VITT, or with the development of severe COVID-19 disease [107].

## Conclusions

It is important to stress that at the time of writing, a definitive causal association between the use of adenovirus-based COVID-19 vaccines and extremely rare reports of thrombosis with thrombocytopenia has not been established. Nevertheless, safety statements have been updated to raise awareness of the potential occurrence of these very rare side effects within 5–20 days after vaccination with adenovirus vector-based anti-SARS-CoV-2 vaccines. Importantly, due to the strong similarity of VITT with HIT, heparin should not be used to treat this syndrome. Since CVST is more prevalent in younger individuals, some countries including the UK have restricted the use of these vaccines to specific age groups as a countermeasure where alternative vaccine formulations are available.

Further research may identify possible mechanisms to modify these adenovirus vectors to reduce the occurrence of these rare side effects. For example, polyethylene glycol-modification of the Ad5 vector can prevent the induction of thrombocytopenia by reducing interactions between platelets and endothelial cells [108]. However, this should be considered with caution as polyethylene glycol can cause anaphylactic reactions in some individuals [109], and its presence in the mRNA vaccines may have been a contributing factor to the rare but serious instances of anaphylaxis after injection with these vaccines [110]. Modifications to the Ad5 capsid fibre protein might also reduce the induction of thrombocytopenia, as has been shown in mice infected with a replication-competent Ad5 strain [111]. Similarly, thrombocytopenia after intravenous Ad5 vector injection was prevented by the inclusion of mutations in the adenovirus that block its ability to bind to CXADR on host cells and instead redirect cell entry via integrin α(v)β(6) [112]. Reducing the EDTA concentration in the vaccine carrier solution could help reduce capillary leakage and endothelial cell damage around the injection site, and by doing so, reduce the acute inflammatory response encountered after vaccination [57]. Finally, halving the dose of for the first inoculation has been put forward as a way to reduce the risk of clotting outcomes and also diminish other more common side effects [113].

These extremely rare cases of apparent VITT should not distract us from the overwhelming benefit of vaccination to both the recipient and the wider population by limiting SARS-CoV-2 infection rates, and reducing the incidence of serious COVID-19 disease, hospital admissions, and deaths. Current studies will also determine whether vaccination similarly reduces the incidence and impact of ‘long-COVID’. Due to their ability to be stored at standard refrigerator temperatures, these adenovirus-based vaccines will play important roles in vaccinating harder to reach communities and those in less developed nations across the globe. This is especially the case for the Ad26.COV2.S vaccine, as just one injection is required [18]. These vector-based vaccines are also ideally suited for rapid modification in order to create adapted vaccines in response to novel SARS-CoV-2 variants of concern.

## Data Availability

Data sharing is not applicable to this article as no new data were created or analysed in this study.

## Abbreviations

Ab: Antibody
Ad26.COV2.S: Johnson & Johnson/Janssen adenovirus-based COVID-19 vaccine
BNT162b2: Pfizer–BioNTech mRNA-based COVID-19 vaccine
ChAdOx1 nCov-19: Oxford-AstraZeneca adenovirus-based COVID-19 vaccine
C3: Complement component 3
C4: Complement component 4
CR2: Complement receptor 2
COVID-19: Disease caused by infection with severe acute respiratory syndrome corona virus 2
CXADR: Coxsackie-adenovirus receptor
CXCL4: Chemokine (C-X-C motif) ligand 4
CVST: Cerebral venous sinus thrombosis
FcγRIIa: Fc fragment ef IgG receptor IIa
GAG: Glycosaminoglycan
Gam-COVID-Vac: Gamaleya adenovirus-based COVID-19 vaccine
HIT: Heparin-induced thrombocytopenia
IL-8: Interleukin-8
MERS: Middle Eastern Respiratory Syndrome
MHRA: Medicines and Healthcare products Regulatory Agency
mRNA-1273: Moderna mRNA-based COVID-19 vaccine
NETs: Neutrophil extracellular traps
PF4: Platelet factor 4
SARS-CoV-2: Severe acute respiratory syndrome corona virus 2
VITT: Vaccine-induced immune thrombotic thrombocytopenia
vWF: von Willebrand factor
